# Protein kinase C δ signaling is required for dietary prebiotic-induced strengthening of intestinal epithelial barrier function

**DOI:** 10.1038/srep40820

**Published:** 2017-01-18

**Authors:** Richard Y. Wu, Majd Abdullah, Pekka Määttänen, Ana Victoria C. Pilar, Erin Scruten, Kathene C. Johnson-Henry, Scott Napper, Catherine O’Brien, Nicola L. Jones, Philip M. Sherman

**Affiliations:** 1 Cell Biology Program, Research Institute, Division of Gastroenterology, Hepatology and Nutrition, Hospital for Sick Children, Toronto, Ontario, Canada; 2 Department of Laboratory Medicine and Pathobiology, Faculty of Medicine, University of Toronto, Toronto, Canada; 3 Vaccine and Infectious Disease Organization, University of Saskatchewan, Saskatoon, Saskatchewan, Canada; 4 Department of Biochemistry, University of Saskatchewan, Saskatoon, Saskatchewan, Canada; 5 University Health Network, University of Toronto, Toronto, Ontario, Canada; 6 Departments of Paediatrics and Physiology, University Toronto, Toronto, Ontario, Canada; 7 Department of Nutritional Sciences, University of Toronto, Toronto, Canada; 8 Faculty of Dentistry, University of Toronto, Toronto, Ontario, Canada

## Abstract

Prebiotics are non-digestible oligosaccharides that promote the growth of beneficial gut microbes, but it is unclear whether they also have direct effects on the intestinal mucosal barrier. Here we demonstrate two commercial prebiotics, inulin and short-chain fructo-oligosaccharide (scFOS), when applied onto intestinal epithelia in the absence of microbes, directly promote barrier integrity to prevent pathogen-induced barrier disruptions. We further show that these effects involve the induction of select tight junction (TJ) proteins through a protein kinase C (PKC) δ-dependent mechanism. These results suggest that in the absence of microbiota, prebiotics can directly exert barrier protective effects by activating host cell signaling in the intestinal epithelium, which represents a novel alternative mechanism of action of prebiotics.

The essential role of the gut microbiota in human health and disease has stimulated increasing interest in therapeutic strategies to alter microbial composition^1^,^2^. One such strategy is the use of dietary prebiotics, which are non-digestible food ingredients that resist absorption in the gastrointestinal tract and are fermented by selected intestinal microbes to stimulate the growth and activities of health-promoting gut microbes, including *Lactobacilli* and *Bifidobacteria*^3^.

Fructans are a group of carbohydrates that fall under the definition of prebiotics and include inulin and fructooligosaccharides (FOS), which are plant-derived polysaccharides comprised of fructose monomers connected via β(2-1) glycosidic bonds linked to a terminal glucose residue^4^. Inulin and FOS differ mainly in chain length, with a degree of polymerization of greater than 10 for inulin and less than 10 for FOS^4^.

The health-promoting benefits of prebiotics have been attributed mainly to indirect effects through either bifidogenic or anti-adhesive properties^2^,^4^,^5^. Prebiotic fermentation by *Lactobacilli* and *Bifidobacteria* results in the production of short-chain fatty acids such as acetate, propionate and butyrate, which create an acidic microenvironment that can antagonize the growth of pathogenic microbes^5^. Furthermore, specific prebiotics can interfere with pathogen adherence by competitively inhibiting the binding of pathogenic microbes to host receptors^6^. For example, enteropathogenic *Escherichia coli* expresses oligosaccharide-binding adhesins that allow the microbe to dock to carbohydrates expressed on the apical epithelial surface^7^. Galactooligosaccharides mimic these binding motifs to inhibit *E. coli* attachment to enterocytes^7^.

Prebiotics may also exert direct effects on the host gut epithelium, but these effects are largely unexplored. This study demonstrates that prebiotics directly act on the intestinal epithelium to elicit specific signaling responses in the absence of microbes. Two commonly used commercial prebiotics, inulin and scFOS, were employed and their effects on barrier function were measured in immortalized gut-derived epithelial cell lines and human intestinal organoids. Addition of both inulin and scFOS maintained epithelial barrier function in the context of epithelial injury caused by a non-invasive human enteric bacterial pathogen, enterohemorrhagic *Escherichia* coli O157:H7 (EHEC). Chemical inhibitors and functional knockdown studies were employed to show that the protective mechanism was due to prebiotic activation of PKCδ. These results indicate that prebiotics *directly* modulate gut homeostasis in a microbe-independent manner.

## Results

### Prebiotics protect the intestinal epithelial barrier from EHEC challenge

**Figure 1:**
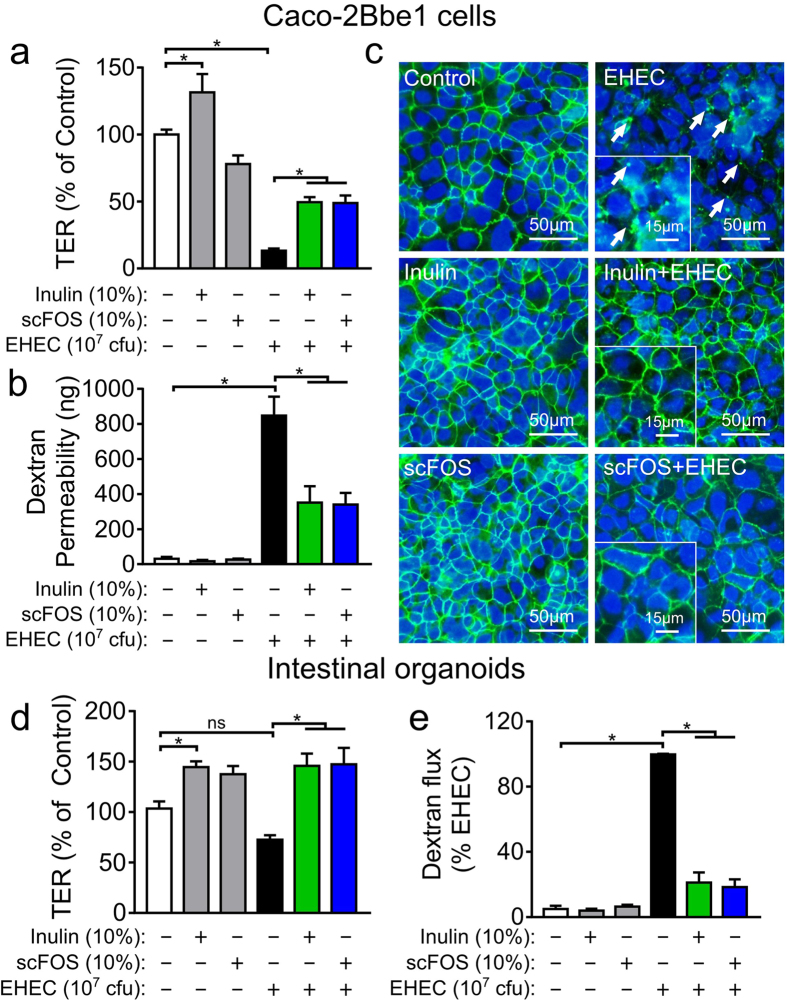
Inulin and scFOS reduce EHEC O157:H7-induced barrier disruption in Caco-2Bbe1 monolayers. (**a**) Polarized Caco-2Bbe1 monolayers were treated in duplicates with inulin or scFOS (10% w/v, 16 h) prior to infection with EHEC (10^7^ CFU, 5 h). Both prebiotics attenuated EHEC-induced decrease in TER and maintained barrier function (n = 5). (**b**) FITC-dextran (10 kDa) was added apically to monolayers and signal intensities of translocation were measured from the basolateral culture media and expressed as absolute dextran quantities in nanograms (n = 5). (**c**) Representative immunofluorescent micrographs of ZO-1 organization in Caco-2Bbe1 monolayers (n = 3). Fixed monolayers were labeled with rabbit anti-ZO-1 (green) and DAPI (blue) for nuclear staining. Arrows show ZO-1 redistribution in the form of diffuse, punctate staining. (**d**) Transwell-grown intestinal organoids were incubated with inulin and scFOS (10%, 16 h) and then challenged with EHEC (MOI:100, 10 h). Both inulin and scFOS increased the TER of EHEC-challenged cells (n = 5–7). (**e**) EHEC challenge was continued for 35 h to induce dextran permeability, and signal intensities of translocation were measured from the basolateral culture media and expressed as percentage of EHEC-control (n = 3). Data are expressed as means ± SEM, and tested using ANOVA with Bonferonni post-hoc testing, *P < 0.05.

To determine barrier function, we measured the transepithelial electrical resistance (TER) of Caco-2Bbe1 monolayers in response to inulin and scFOS with or without EHEC challenge. In the unchallenged state, only inulin, but not scFOS, increased the TER of Caco-2Bbe1 cells (Fig. 1a). In the challenged state, EHEC decreased TER to 13.3 ± 1.6% of baseline, but pre-incubation with either inulin or scFOS attenuated the EHEC-induced decline in TER resulting in post-challenge TER of 49.9 ± 3.7% and 48.9 ± 5.6% for inulin and scFOS, respectively (Fig. 1a). This protection is time-dependent as shown in the time-TER response (Supplementary Fig. 1a) where the effect was maximal at 4–5 h but abolished by 8 h of EHEC exposure – an observation congruous to what we have described previously^8^. Using this optimal 5 h window, we confirmed TER results using a fluorescein-labeled isothiocyanate (FITC) dextran permeability assay, where both inulin and scFOS-treated cells exhibited reduced dextran permeability after the 5 h EHEC challenge (Fig. 1b). We next evaluated whether enhanced intercellular TJ integrity is responsible for the barrier protective effects of prebiotics. The cellular response to EHEC challenge seen using immunofluorescence microscopy includes the transformation of zona occluden-1 (ZO-1) from continuous, circumferential staining to diffuse, punctate staining (Fig. 1c)^9^. However, the presence of either inulin or scFOS prevented EHEC-induced redistribution of ZO-1. In comparison, unchallenged but prebiotic-treated cells displayed morphology comparable to untreated control cells.

To compliment the barrier protective effects of inulin and scFOS, we performed the same assays on *ex vivo* cultured duodenal organoids derived from three individual patients. Unlike cancer-derived epithelial cell lines, organoids are derived from primary stem cells and consist of a heterogeneous cell population with cell-type specific markers which were unaltered by the presence of inulin or scFOS (Supplementary Fig. 1b).When grown on Transwell filter supports, the intestinal organoids form a two-dimensional monolayer with clear cell border demarcations and TJ (ZO-1) expression (Supplementary Fig. 1c). Under these conditions, EHEC challenge did not impact the TER of human intestinal organoids (Fig. 1d, P > 0.05) but significantly increased FITC-dextran permeability (Fig. 1e). Similar to Caco-2Bbe1 cells, pre-incubation of organoids with either inulin or scFOS significantly increased TER during EHEC challenge (Fig. 1d) and prevented the EHEC-mediated increase in FITC dextran permeability (Fig. 1e). Interestingly, in the unchallenged state, scFOS increased TER even though this was not seen in the Caco-2Bbe1 cells. Taken together, these findings indicate a generalized protective response whereby prebiotics directly enhance epithelial barrier integrity to protect against pathogen-induced barrier disruption in physiological models of human gut epithelia.

### Prebiotics regulate TJ protein and mRNA levels

**Figure 2:**
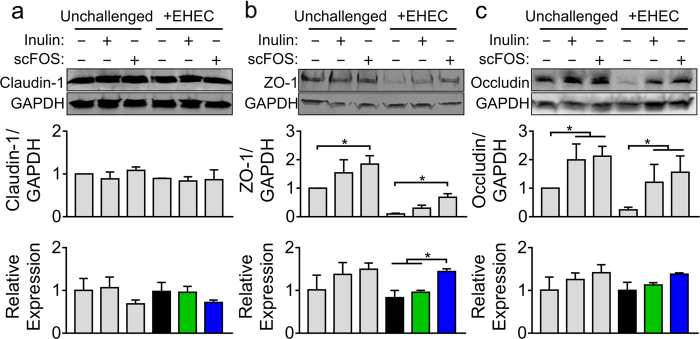
Inulin and scFOS regulate TJ protein expression. (**a**) Caco-2Bbe1 monolayers pretreated with inulin or scFOS (10% w/v, 16 h) and challenged with EHEC (10^7^ CFU, 5 h) showed no significant changes in claudin-1 mRNA (n = 4) and protein levels (n = 3). (**b**) scFOS increased ZO-1 mRNA levels in EHEC-challenged cells and prevented the EHEC-induced decline in ZO-1 protein levels (n = 4) compared to inulin. (**c**) Neither inulin nor scFOS affected occludin mRNA expression (n = 4), but both significantly increased protein levels in both EHEC-challenged and non-challenged cells (n = 4). Relevant gel bands were cropped from the original blots. Densitometry ratios were measured using GAPDH as the loading control. All values are expressed as means ± SEM. ANOVA with Bonferronni post-hoc testing, *P < 0.05.

To determine whether the enhanced epithelial barrier integrity involved changes in TJ expression, both mRNA and protein levels of claudin-1, ZO-1 and occludin were measured. Neither prebiotic altered claudin-1 mRNA or protein levels (Fig. 2a). ScFOS, but not inulin, increased ZO-1 mRNA expression in EHEC challenged cells and increased ZO-1 protein levels in both unchallenged and challenged cells (Fig. 2b). Inulin and scFOS also increased protein levels of occludin in the absence or presence of EHEC, but without altering occludin mRNA expression (Fig. 2c). This result suggests that prebiotics may directly alter select TJ expressions to affect epithelial barrier function.

### Prebiotics impact the host kinome response

**Figure 3:**
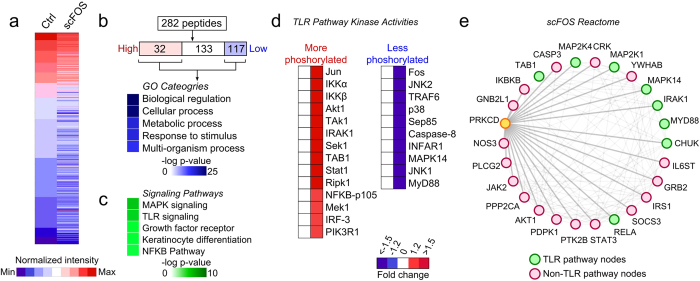
ScFOS alters host kinase activities across multiple immune regulatory pathways. (**a**) Heat map displaying the modulation of kinome activities in Caco-2Bbe1 cells treated with scFOS. (**b**) Gene enrichment analysis of differentially phosphorylated kinases in response to scFOS clustered into gene ontology functional categories and (**c**) pathways from BIOCARTA database (only the five most highly enriched are shown). (**d**) Heatmap comparing phosphorylation levels of scFOS-mediated kinases involved in TLR pathways. (**e**) Interaction network of modulated kinases in response to scFOS (both physical and pathway interactions are displayed). Ctrl denotes control.

To investigate the potential mechanisms mediating prebiotic modulation of barrier function and TJ expression, we measured the effects of scFOS on the host kinome using a 282-peptide immune array to screen for potential pathways^10^,^11^. As depicted in Fig. 3a, in 15 minutes of exposure, scFOS induced global shifts in the kinome activity of Caco-2Bbe1 cells. Of the 282 peptides surveyed, scFOS differentially phosphorylated 149 peptides by a fold change of greater than 1.2. To interrogate the function of these kinases, we performed gene enrichment analysis and identified specific clusters of gene ontology categories (Fig. 3b) and immune pathways that were modified (Fig. 3c). Notably, in line with recent reports suggesting glycan modulations of Toll-like Receptors (TLRs)^12^, kinases involved in MAPK and TLR were highly annotated in our analysis. Inspection of specific kinases involved in TLR signaling revealed 24 differentially phosphorylated kinases (Fig. 3d) whose specific roles in the TLR pathway are presented in Supplementary Fig. 2.

One of the ways in which TLR pathways regulate epithelial barrier is the activation of protein kinase C (PKC)^13^. The PKC family of serine/threonine kinases has 12 known isoforms that activate various downstream pathways such as mitogen-activated protein kinases (MAPKs)^14^,^15^, and participates in a variety of cellular processes^14^,^16^,^17^. Select PKC isoforms, such as α and δ, are highly expressed in the intestinal epithelium isoforms and are involved in the assembly of intercellular TJ^13^,^17^. To assess the possible connections between PKC and TLR pathways, we queried all 149 modulated peptides and interrogated their protein interactions with PKC isoform δ using GeneMania^18^. This analysis revealed 26 peptides (Fig. 3e), 24 of which directly interact with PKCδ in extensive physical or pathway interactions. The functional pathway most highly enriched in this data set was TLR signaling. Taken together, these findings suggest that scFOS induces a TLR-related response in intestinal epithelial cells that may involve a PKC-mediated mechanism.

### Inulin and scFOS activate specific PKC isoforms in intestinal epithelial cells

**Figure 4:**
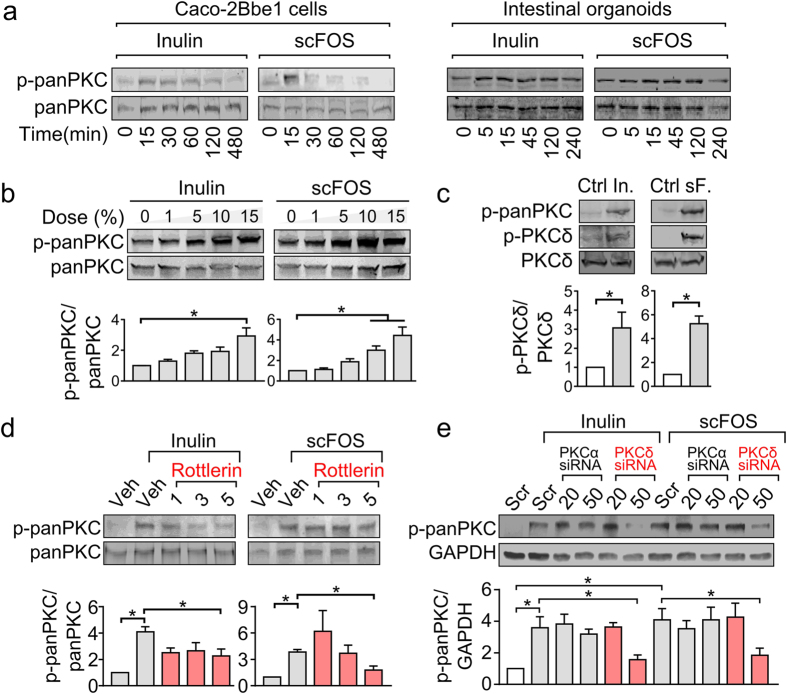
Inulin and scFOS induce activation of host PKC signaling in a time- and dose-dependent manner. (**a**) Caco-2Bbe1 monolayers (n = 3) and intestinal organoids (n = 3) were treated with inulin (10% w/v) or scFOS (10% w/v) for the indicated time periods (15–480 min) and blotted for phospho-panPKC. (**b**) Caco-2Bbe1 monolayers were treated with varying doses (0–15% w/v) of inulin or scFOS at 15 minutes of exposure (n = 4). (**c**) Inulin (10% w/v) and scFOS (10% w/v) induced phosphorylation of PKCδ after 15 min exposure in Caco-2Bbe1 cells (n = 4). (**d**) In Caco-2Bbe1 cells pretreated with PKCδ inhibitor rottlerin (1, 3 and 5 μM, 1 h), inulin and scFOS showed diminished phospho-panPKC response at 5 μM (n = 4). Cells that were not treated with rottlerin were exposed to vehicle control (0.5% DMSO solubilized in Caco-2Bbe1 medium). (**e**) Stimulation of cells with either inulin or scFOS (10%, 15 min) no longer induced a phospho-panPKC response in the presence of PKCδ siRNA compared to prebiotic-treated cells with PKCα siRNA (n = 4). Cells that were not treated with PKCα or δ siRNA were treated with scrambled siRNA provided by manufacturer in equal volumes of OPTI-MEM and Oligofectamine. Relevant gel bands were cropped from the original blots. All values are presented as means ± SEM, and tested using ANOVA with Bonferroni post-hoc testing, *denotes P < 0.05. (Ctrl, control; veh, vehicle; scr, scrambled).

To check whether PKC is involved in prebiotic modulation of barrier function and TJ expression, we measured PKC activity in prebiotic-treated Caco-2Bbe1 cells and intestinal organoids. Using a phospho-panPKC antibody that detects phosphorylation in a broad range of isoforms, we found that inulin and scFOS both induced panPKC phosphorylation in a time- (Fig. 4a) and dose- (Fig. 4b) dependent manner, as well as activating downstream P38 and P44/42 MAPK (ERK1/2) pathways (Supplementary Fig. 3a,b). We also assessed these signaling events in 2D-grown intestinal organoids (Fig. 4a, Supplementary Fig. 3c). While both inulin and scFOS induced MAPK signaling, in contrast to the effects observed using Caco-2Bbe1 cells, the panPKC phosphorylation response to inulin or scFOS was not evident using organoids.

Next, to identify which PKC isoform(s) was targeted by prebiotics, we determined the levels of phosphorylated isoforms α and δ. Following 15 min of exposure, inulin and scFOS induced phosphorylation of PKCδ (Fig. 4c), but not PKCα (Supplementary Fig. 3d). This finding was confirmed using a PKCδ-specific inhibitor rottlerin, which prevented the panPKC phosphorylation induced by either inulin or scFOS (Fig. 4d). Similarly, panPKC phosphorylation was also attenuated by transfecting Caco-2Bbe1 cells with PKCδ siRNA, but not with PKCα siRNA (Fig. 4e, Supplementary Fig. 3e,f). To ensure PKCα was efficiently knocked down by the construct, we measured PKCα levels as shown in Supplementary Fig. 3g. To control for the general effects of osmotic stress, we also compared the PKCδ activation of scFOS (10% w/v) using iso-osmotic controls 10% w/v maltodextrin and 10% w/v lactose (Supplementary Fig. 3h). As shown in the immunoblots, neither of the iso-osmotic controls induced PKCδ activation, suggesting that PKCδ activation was likely a structure-specific response.

### PKCδ signaling is required for prebiotic protection of intestinal barrier integrity

**Figure 5:**
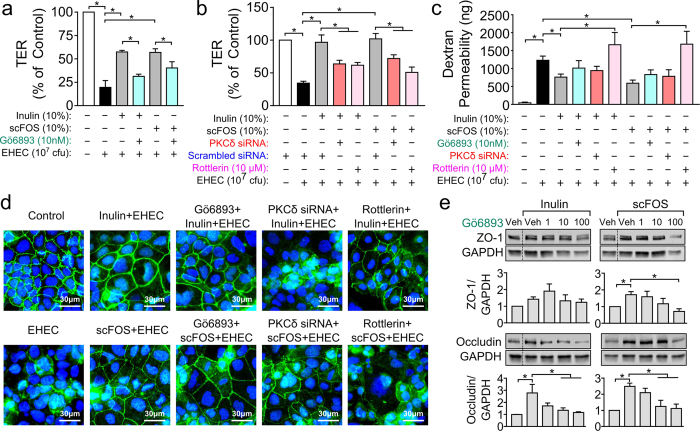
Inhibition of PKC phosphorylation abolishes prebiotic-mediated protection of epithelial barrier function. (**a,b**) In Caco-2Bbe1 cells pretreated with the broad spectrum PKC inhibitor Gö6893 (1, 10, 100 nM, 1 h), rottlerin (1, 3 and 5 μM, 1 h) or PKCδ siRNA, inulin and scFOS lost the capacity to maintain TER following EHEC challenge (n = 4–5). (**c**) Inulin and scFOS failed to reduce EHEC-induced increases in dextran permeability in the presence of PKCδ inhibitor rottlerin, but not Gö6893 or PKCδ siRNA (n = 4). (**d**) Immunofluorescence microscopy of epithelial cells inhibited with Gö6893, rottlerin or PKCδ siRNA showed more disrupted ZO-1 localization following inulin or scFOS exposure, with more punctate staining compared to prebiotic exposure in the absence of inhibitors or PKCδ siRNA (n = 3). (**e**) PKC inhibition with Gö6893 blocked prebiotic-mediated upregulation of ZO-1 and occludin protein expression (n = 4). Cells that were not treated with Gö6893 or rottlerin were treated with vehicle control (0.5% DMSO solubilized in Caco-2Bbe1 medium), while for siRNA experiments untreated cells were transfected with scrambled siRNA provided by manufacturer in equal volumes of OPTI-MEM and Oligofectamine. Relevant gel bands were cropped from the original blots. Dotted line indicates splicing of two regions together within the same blot. All data are expressed as means ± SEM, and tested using ANOVA with Bonferonni post-hoc testing, *P < 0.05.

To investigate the link between prebiotic barrier effects and PKCδ signaling, we tested the impact of inhibiting PKCδ on the prebiotics’ ability to maintain TER following EHEC challenge. As shown in Fig. 5a,b, prebiotic attenuation of the EHEC-induced drop in TER was blocked by treating cells with the panPKC inhibitor Gö6893, rottlerin or PKCδ siRNA. Rottlerin-treated cells demonstrated greater dextran permeability (Fig. 5c). Loss of TJ ZO-1 organization was also observed in rottlerin-treated cells but not in cells with Gö6893 (Fig. 5d). Next, to compare TJ expression effects, we blocked Caco-2Bbe1 cells with Gö6893 prior to prebiotic exposure and measured ZO-1 and occludin protein levels. Gö6893 (100 nM) prevented scFOS-induced ZO-1 expression, while increases in occludin expression induced by either prebiotic was also prevented by 10 and 100 nM Gö6893 (Fig. 5e). Caco-2Bbe1 cells treated with Gö6893 alone at 1, 10 and 100 nM did not alter expression levels of either ZO-1 or occludin (Supplementary Fig. 4). Taken together, these results indicate that PKCδ activity is required for the protective effects of inulin and scFOS on epithelial barrier integrity.

## Discussion

Although it is generally recognized that prebiotics affect human health by altering the gut microbiota^5^, much less is known about the extent of direct effects on the host mucosal surface^2^. Previous studies have shown that β(2–1) fructans increase the TER of T84 monolayers, which was hypothesized to be due to PKC signaling^19^. In the present work, we tested this possibility to provide the first evidence for a direct mechanism whereby prebiotics, agents believed to be mostly inert to the host, activate the PKCδ signal transduction pathway to regulate TJ integrity and mitigate the deleterious effects of EHEC. These results demonstrate a specific and direct host-nutrient interaction and elucidate a novel mechanism whereby prebiotics maintain gut homeostasis to protect the host against challenge by enteric pathogens.

In this study, we showed that the prebiotics inulin and scFOS maintain TER and decrease dextran flux in an EHEC injury model by upregulating occludin and ZO-1 expression. TJs encircle and anchor adjacent cells, and depletion of their expression disrupts the ability of epithelial cells to form a barrier and maintain TER^20^. Prebiotics reduce gut permeability and facilitate claudin-3 assembly and expression^21^,^22^, but the underlying mechanisms have remained unclear. Cani and colleagues previously found that prebiotics increased the expression of both ZO-1 and occludin in mice via a microbe-dependent mechanism^21^, but the possibility of direct non-microbial mechanism has also been suggested^23^. We previously identified that atypical PKC isoforms are involved in the signaling responses to EHEC^24^, we extend these findings by now showing PKC signaling can also be triggered by exposure to dietary prebiotics to alter epithelial barrier and TJ function. This observation was validated through PKC loss-of-function experiments, which abolished the protective effects of prebiotics on epithelial barrier integrity. These findings are supported by previous studies showing that activation of the PKCδ pathway increases TER and promote assembly of ZO-1^13^. Taken together, these results support the emerging theory that in addition to microbial interactions, prebiotics can impact gut homeostasis via direct mechanisms on host signaling pathways.

Traditionally, the antimicrobial properties of prebiotics are attributed to their role as decoy receptors. Bacterial adhesins bind to sugar motifs expressed on the cell membrane and prebiotics bearing such motifs interfere with binding^4^. However, using immunostaining we previously observed that neither inulin or scFOS affected the formation of attaching and effacing lesions in EHEC-challenged cells^8^, indicating that physical inhibition was not a major factor in barrier protection. Additionally, our results also demonstrated that changes in TJ expression and TER occurred in the absence of EHEC challenge, which again suggests that the protective effects are independent of bacteria interference. However, our results do not preclude the possibility that prebiotics may also regulate the expression of EHEC virulence factors. In *Pseudomonads,* for example, FOS reduced the expression of exotoxin A and inhibited NF-κB activation^25^. Whether inulin and scFOS can regulate the expression of EHEC virulence factors remains an open question. Furthermore, it is not known whether prebiotics induce the expression of antimicrobial peptides such as defensins through the involvement of TLRs. Although we have previously found that neither inulin or scFOS affects the proportion of viable EHEC at 5 h^8^, further characterization of these effects on bacterial cells is warranted.

Downstream PKC phosphorylation is implicated in ligand activation of several receptors, including G-protein Coupled Receptors (GPCRs) and TLRs^13^,^15^,^26^,^27^. Similar to the PKC kinetics seen in these pathways^13^,^27^, both inulin and scFOS induced maximal PKC phosphorylation and kinome responses at 15 min, suggesting that prebiotics directly activate membrane receptors to elicit signal transduction. The observation that scFOS induced kinome activities across several pathways suggests that PKCδ activation is not a singular event, but rather occurs as part of a global shift in signaling induced by prebiotics. Interestingly, we found kinase activities were significantly enriched in TLR pathways, many of which also interact with PKCδ in pathway and physical interactions. These kinase activities align with our previous work on the expression of inflammatory cytokines/chemokines^8^, which showed that in the absence of EHEC, scFOS changed the expression of TLR-modulated genes including *IL-10, TNF-α, CXCL-8* and *CXCL-1*. Taken together, these results support a view that prebiotics may be directly activating TLR signaling to alter cytokine/chemokine expressions and the integrity of the epithelial barrier. This is also in line with multiple reports showing that prebiotics induce cytokine and chemokine production in monocytes and intestinal epithelial cells in a TLR2- or TLR4-dependent fashion^12^,^19^,^28^,^29^,^30^,^31^. However, whether PKCδ is the only PKC isoform or the only signal transduction pathway affected is still unclear. One major caveat in these studies is the non-targeted effects of PKCδ siRNA and chemical inhibitors. RNA interference using PKCδ siRNA could produce non-specific effects such as cross-reactivity with other isoforms. Similarly, chemical inhibitors could have non-specific effects and induce cytotoxicity. In addition, as seen in the kinome analyses, numerous signaling mediators were activated by scFOS. Due to the known promiscuity of some of these effector kinases, it is likely that other signal transduction effects are also involved. Careful characterization of these downstream signaling events and their particular relations with tissue inflammation should be the subject of future research.

One interesting observation in our study is the specificity of prebiotic effects, which are not only glycan-specific, but also cell type-specific^32^. For instance, although EHEC-challenged Caco-2Bbe1 cells and intestinal organoids show consistency in macromolecule fluxes and TER responses, the TER response to scFOS and the extent of panPKC phosphorylation in unchallenged cells was different in the two models. This was not too surprising given the differences in cell types: Caco-2Bbe1 cells are homogenous enterocytes derived from a colorectal cancer, while organoids were derived from non-cancerous duodenal biopsies and contained multiple primary cell types. Prebiotics could well elicit differential effects on tissues across various regions of the intestinal tract, and thus it is likely that diverse mechanisms of action could be responsible for the barrier function effects seen when using transformed cell lines versus primary intestinal organoids. Nevertheless, the data presented herein with the complementary gut model systems demonstrate that in the absence of a functional microbiome, prebiotics do exert direct protective effects on the integrity of the epithelial barrier to mitigate disruption triggered by exposure to an enteric microbial pathogen.

In summary, the findings of this study show that prebiotics modulate host cell signaling to promote epithelial barrier integrity by direct effects on the intestinal mucosa. Such a mechanism is seen in plants where sugars are the main signaling molecules in immune pathways, and act as priming agents to confer resistance against microbial invasion^33^,^34^. During fungal infection, for instance, plants release the polysaccharide oligogalacturonide from cell walls to facilitate an innate immune response by activating wall-associated kinases analogous to mammalian TLRs^35^,^36^. Similar mechanisms may well be relevant in humans. Today, a prebiotic is a widely recognized functional food with reported benefits in conditions such as obesity^37^,^38^ and GI disorders^4^, but the underlying mechanisms of action are unclear. By demonstrating prebiotic fibers can act as direct signaling molecules in the host intestine, the ramifications are manifold in shaping current thinking about the benefits of dietary fiber that were previously unrecognized.

## Methods

### Prebiotics

Inulin (Quadra, Burlington, Canada) and scFOS (Nutraflora^®^, Nutrition GTC, Golden, CO) are inulin-type fructan polymers that differ in chain lengths, where scFOS contains 2–9 degrees of polymerization (DP) of fructose monomers while inulin has a DP of >10^4^. To treat cells, inulin and scFOS (1–15% w/v) were solubilized in pre-warmed, antibiotic-free Dulbecco’s modified Eagle medium for Caco-2Bbe1 cells, or 2D culture medium for intestinal organoids (formulation listed below).

### Bacterial culture

EHEC was grown from a frozen stock on Columbia 5% blood agar plates (BBL) overnight at 37 °C. Cultures were prepared from individual colonies in Penassay broth (Gibco, Waltham, MA) and grown statically at 37 °C. Overnight cultures were subcultured into fresh Penassay broth for 3 h, and then added onto cell monolayers at a multiplicity of infection (MOI) of 100:1.

### Cell culture and transepithelial electrical resistance

Caco-2Bbe1 cells, a differentiated Caco-2 subclone which attains faster confluency, were purchased from American Type Culture Collection and grown using cell culture conditions as previously described^8^,^24^. Cells were harvested with trypsin-EDTA and seeded onto 6.5-mm Transwell filter supports (~1 × 10^5^ cells/insert, Corning, Mississauga, Canada) and maintained for 3–4 d until TER reached >800 Ω cm^−2^ measured using chopstick electrodes (Millipore, Etobicoke, Canada)^9^,^39^. The apical medium was replaced with prebiotic (10% w/v, 16 h), and then challenged with EHEC (5 h). Data are expressed as percentages of TER at baseline relative to untreated wells.

### Generation and culturing of human intestinal organoids

Ethics approval for this study was obtained from the Ethics Committee of the University Health Network, Toronto, Canada (protocol #15-9437). Informed consent was obtained from all patients prior to their participation. All methods performed in the study were carried out in accordance with the approved guidelines and regulations, and signed informed written consent was collected from all participants prior to participation in the study. Duodenum tissue resections from patients undergoing pancreatico-duodenectomy (Whipple procedure) were used to culture human intestinal organoids. As previously described^40^, overlying mucus and the underlying muscular layer were removed and the tissue was cut into 20–30 small pieces (2 mm^2^) and incubated in 5 mM EDTA in AdvanceSTEM ES Qualified DPBS (HyClone, Thermo Fisher, Walktham, MA) for 1 h at 4 °C on an orbital shaker. Crypts were released by mechanical shaking in 1% fetal bovine serum (FBS) in DPBS and isolated by centrifugation (500 *g*, 10 min). Crypts were resuspended in Matrigel (BD Biosciences, San Jose, CA) at approximately 250 crypts per 25 μL of Matrigel, plated into individual wells of a 24-well plate and placed at 37 °C for 5 min to polymerize. After Matrigel polymerization, embedded crypts were overlayed with organoids culture medium (described in Supplemental information).

Organoids were maintained by replacing culture medium 2–3 days and passaging 5–10 days at 1:2–4. For passaging, culture medium was aspirated and Matrigel dissolved in ice-cold basal medium (advanced DMEM/F12 supplemented with 10 mM HEPES, 1% GlutaMAX and 1% pen/strep). Organoids were transferred to a 15 mL conical tube and mechanically sheared 10–20 times with a fire-polished glass Pasteur pipette. Sheared organoids were centrifuged and resuspended in cold Matrigel.

### Primary human intestinal monolayers

To form intestinal epithelial monolayers, 400–500 human organoids from a 4–6 day old culture (2–3 wells of a 24-well plate per Transwell) were dissociated with mechanical shearing and resuspended in TrypLE Express Enzyme (Thermo Fisher) for 3 min at 37 °C. Trypsinized organoids were sheared again to dissociate into single cells and TrypLE inactivated with basal medium containing 10% FBS. Epithelial cells were resuspended in organoid culture medium and then seeded onto Transwell filter supports coated with 1:40 Matrigel in PBS (~1 h at 37 °C). Cells were left to adhere overnight and organoid medium replaced with 2D culture medium (advanced DMEM/F12 supplemented with 10% FBS, 1% N_2_, 2% B27, 50 ng/mL epidermal growth factor, 1 μM TGFβi, 10 mM nicotinamide and 10 μM Y-27632). Confluent monolayers formed 4–5 days after seeding.

### FITC-dextran flux

Paracellular permeability was measured using fluoresceinated dextran (10 kDa, Life Technologies, Thermo Fisher). Briefly, following final TER measurements (5 h for Caco-2Bbe1 cells, 35 h for intestinal organoids), apical compartments were washed with PBS and replaced with solution containing 100 μg/ml FITC-dextran dissolved in DMEM, and basolateral chambers replaced with fresh DMEM. After 5 h incubation at 37 °C, aliquots from the basolateral compartments were collected and signal intensities measured at 700 nm (emission) using an infrared imaging system (Odyssey^®^, LI-COR Biosciences, Lincoln, NE). Quantified fluorescence signal intensities were converted to absolute dextran quantities (in nanograms) by using a serial dilution curve.

### Immunofluorescence microscopy

Polarized cell monolayers were fixed in paraformaldehyde, permeabilized in 0.1% Triton X-100, blocked with 3% bovine serum albumin (Sigma, St. Louis, MO) and incubated with rabbit anti-ZO-1 antibody (Life Technologies) and goat Alexa fluor 488-conjugated anti-rabbit IgG antibodies (Life Technologies) as described previously^8^. Cells were then mounted onto glass slides using ProLong mounting solution (Life Technologies) and imaged with a Leica DMI6000B fluorescence microscope and companion DFC 360FX camera (Leica Microsystems, Concord, Canada) with 20x magnification.

### Immunoblotting

Cell monolayers were lysed, as previously described^24^, and lysates separated using SDS-PAGE followed by transfer onto nitrocellulose membranes (BioTrace NT, Ann Arbor, MI). Descriptions of primary antibodies used can be found in the supplemental information. Secondary antibodies (IRDye 680 goat anti-rabbit IgG or IRDye 800 goat anti-mouse IgG) were purchased from Rockland (Limerick, PA). Immunoblots were analyzed using an Odyssey imaging system (LI-COR Biosciences). Densitometry ratios were calculated using ImageJ (NIH, Bethesda, MD). Image intensity of bands was normalized to either GAPDH or non-phosphorylated bands, and expressed as a ratio of the control, which was set at 1.

### Transfections

PKCα, PKCδ and scrambled siRNAs were obtained from Santa Cruz (Dallas, TX) and transfected using Oligofectamine 2000 (Thermo Fisher), in accordance with manufacturer’s protocol. Briefly, Caco-2Bbe1 cells were seeded onto 24-well plates (~5 × 10^4^ cells/well) and transfected with siRNA oligomers complex dissolved in OPTI-MEM (Life Technologies) at 10, 20 and 50 pmoles for 48 h prior to experiments. Control cells were treated with scrambled siRNA using equal amounts of OPTI-MEM and Oligofectamine reagents. Verification of siRNA knockdown efficiency was performed by western blotting for PKCα and PKCδ.

### Pharmacological inhibitors

Broad spectrum PKC inhibitor Gö6983 and PKCδ-specific inhibitor rottlerin were purchased from Calbiochem (San Diego, CA). Gö6983 inhibits various isoforms of PKC: PKCα (7 nM), PKCβ (7 nM), PKCγ (6 nM), PKCδ (10 nM) and PKCζ (60 nM). Inhibitors were dissolved in DMSO and suspended in tissue culture medium at 1, 10 or 100 nM and 1, 3 or 5 μM for Gö6983 and rottlerin, respectively. Inhibitors were added onto cells for 1 h prior to prebiotic addition without removal or washing. Control cells were treated with vehicle (medium containing 0.5% DMSO). Phorbol myristate acetate (PMA), a chemical inducer of PKC^41^, was used as a positive control to verify inhibition of PKC phosphorylation by each of the inhibitors.

### Kinome array

As described previously^10^,^42^, Caco-2Bbe1 cells grown in 6-well plates (~1 × 10^6^ cells) were incubated with or without 10% w/v scFOS for 15 min. Cells were then lysed with lysis buffer containing 20 mM Tris-HCl [pH 7.5], 150 mM NaCl, 1 mM EDTA, 1% Triton, 2.5 mM sodium pyrophosphate, 1 mM Na_3_VO_4_, 1 mM NaF, 1 μg/ml leupeptin, 1 g/ml aprotinin, 1 mM PMSF (Sigma). Sample lysates were incubated on the peptide array for 2 h at 37 °C, washed, and submerged in phospho-specific fluorescent ProQ Diamond phosphoprotein stain (Thermo Fisher). Arrays were washed three times to remove excess stain with 20% acetonitrile before a final water wash and prior to being dried and read using a GenePix Professional 4200 A microarray scanner (MDS Analytical Technologies, Toronto, Canada).

Spot intensity signals were collected as mean pixel intensity using local feature background subtraction calculation with default scanner saturation levels. Intensity signals for replicate spots of each peptide were processed to generate a heat map using peptide kinome array-specific analysis methods previously reported^10^. For analysis of gene ontology functional categories and pathway enrichments, peptides were uploaded onto DAVID and GeneMania for functional annotation and interaction analysis. Heatmap and pathways were generated using GenePattern and Ingenuity Pathway Analysis.

### qRT-PCR

Total RNA was isolated using TriZOL (Thermo Fisher) following the manufacturer’s protocol and reverse-transcribed to cDNA using iScript cDNA synthesis kit (Bio-Rad, Mississauga, Canada). qPCR procedure is described in the supplemental information.

### Statistics

Results are expressed as means ± SEM derived from at least 3 independent experiments. All statistics were performed using Graphpad Prism 6.0 (Prism) Comparisons of multiple groups were performed using one-way ANOVA with Bonferonni test for *post-hoc* analysis. Pairwise comparisons were undertaken using the unpaired Student’s t-test. P values less than 0.05 were deemed statistically significant.

## Additional Information

**How to cite this article**: Wu, R. Y. *et al*. Protein kinase C δ signaling is required for dietary prebiotic-induced strengthening of intestinal epithelial barrier function. *Sci. Rep.* **7**, 40820; doi: 10.1038/srep40820 (2017).

**Publisher's note:** Springer Nature remains neutral with regard to jurisdictional claims in published maps and institutional affiliations.

## Supplementary Material

Supplemental Methods and Figures
