# Supplementary material for: Protein kinase C δ signaling is required for dietary prebiotic-induced strengthening of intestinal epithelial barrier function
Source: Sci Rep. 2017 Jan 18;7:40820. doi: 10.1038/srep40820 (PMC5241689; doi:10.1038/srep40820)
Supplement: Supplemental Methods and Figures [file srep40820-s1.pdf]

MANUSCRIPT TITLE: Protein kinase C δ signaling is required for dietary prebiotic-induced strengthening of intestinal epithelial barrier function

Authors: Richard Y. Wu[1,2], Majd Abdullah[1], Pekka Määttänen[1], Ana V. Pilar[1], Erin Scruten[3], Kathene C. Johnson-Henry[1], Scott Napper[3,4], Catherine O'Brien[2,8], Nicola L. Jones[1,7], *Philip M. Sherman[1,2,5,6]

[1]Cell Biology Program, Research Institute, Division of Gastroenterology, Hepatology and Nutrition, Hospital for Sick Children, Toronto, Ontario, Canada; [2]Department of Laboratory Medicine and Pathobiology, Faculty of Medicine, University of Toronto, Toronto, Canada; [3]Vaccine and Infectious Disease Organization, University of Saskatchewan, Saskatoon, Saskatchewan, Canada; [4]Department of Biochemistry, University of Saskatchewan, Saskatoon, Saskatchewan, Canada; [5]Department of Nutritional Sciences, University of Toronto, Toronto, Canada; [6]Faculty of Dentistry, University of Toronto, Toronto, Ontario, Canada; [7]Departments of Paediatrics and Physiology, University Toronto, Toronto, Ontario, Canada; [8]University Health Network, University of Toronto, Toronto, Ontario, Canada.

SUPPLEMENTAL INFO

METHODS

qRT-PCR

qRTPCR was performed in a CFX96 C1000 Thermal Cycler (Bio-Rad) using iQ SYBR Green Supermix with 500 ng of template RNA and the following primers (5'-3') were utilized:
ZO-1, GAATGATGGTTGGTATGGTGCG (forward), TCAGAAGTGTGTCTACTGTCCG (reverse);
Claudin-1, AGCTGGCTGAGACACTGAAGA (forward), GAGAGGAAGGCACTGAACCA (reverse);
Occludin TTGGATAAAGAATTGGATGACT (forward), ACTGCTTGCAATGATTCTTCT (reverse);
GAPDH ACCCACTCCTCCACCTTTGAC (forward), CCACCACCCTGTTGCTGTAG (reverse)
β-actin CTGGAACGGTGAAGGTGACA (forward), AAGGGACTTCCTGTAACAATGCA (reverse).

Expression levels were calculated by the $\Delta\Delta C_t$ method and normalized to two reference housekeeping genes (*GAPDH* and *β-actin*).

Human intestinal organoids

Culture medium for intestinal organoid includes advanced Dulbecco's modified Eagle medium/F12 (Thermo Fisher) containing 50% conditioned Wnt3a-medium, 25% conditioned Rspo1-medium and 10% conditioned noggin-medium, supplemented with 1% penicillin/streptomycin, 10 mM HEPES, 1% GlutaMAX, 1% N2, 2% B27 (all from Thermo Fisher), 50 ng/mL epidermal growth factor (R&D Systems), 1 mM *N*-acetyl-cysteine, 10 μM Y-27632, 10 mM nicotinamide, 10 nM Gastrin (all from Sigma-Aldrich) and 1 μM TGFβi (A-83-01; Tocris).

Immunoblotting

Primary antibodies anti-ZO-1, anti-occludin and anti-claudin-1 were purchased from Invitrogen; anti-GAPDH, anti-panPKC, anti-PKCδ, anti-PKCα and anti-phospho-PKCα were purchased from Santa-Cruz; anti-phospho-panPKC (detects α, βI, βII, δ, ε, η and θ isoforms), anti-phospho-ERK 1/2, anti-ERK 1/2, anti-phospho-P38 and anti-P38 antibodies were purchased from Cell Signaling and anti-phospho PKCδ was purchased from Abcam.

SUPPLEMENTAL DATASET

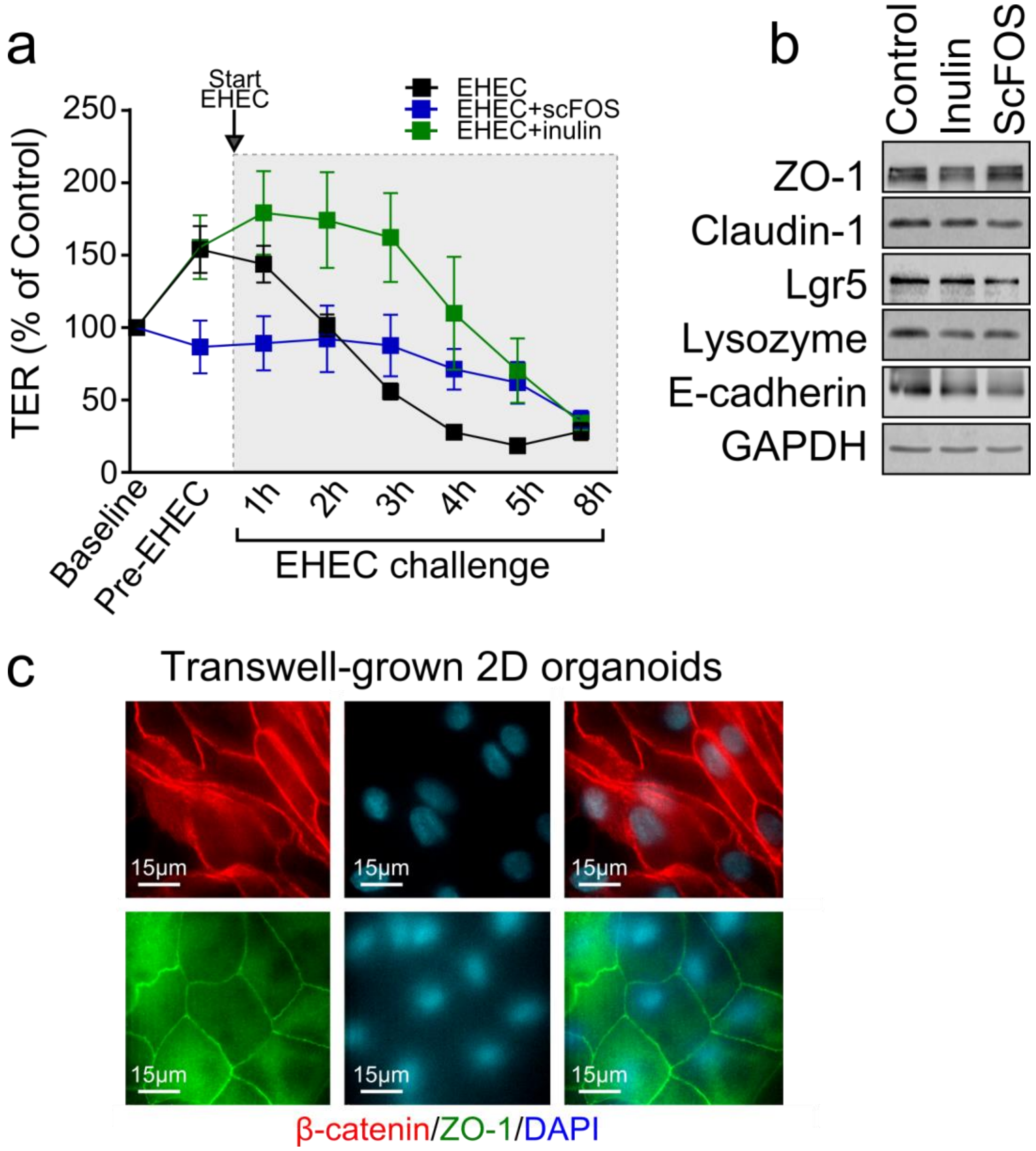

Supplementary Figure 1. Characterization of 2D-grown intestinal organoids. (**a**) TER of Caco-2Bbe1 cells in response to varying exposure durations to EHEC at a MOI of 100 (n=4-6), expressed as means ± SEM. (**b**) 2D intestinal organoids grown in Transwells were immunoblotted for cell type and differentiation markers. (**c**) Immunofluorescence microscopy images of Transwell-grown 2D organoid monolayers stained for ZO-1, β-catenin and DAPI for nuclear stain.

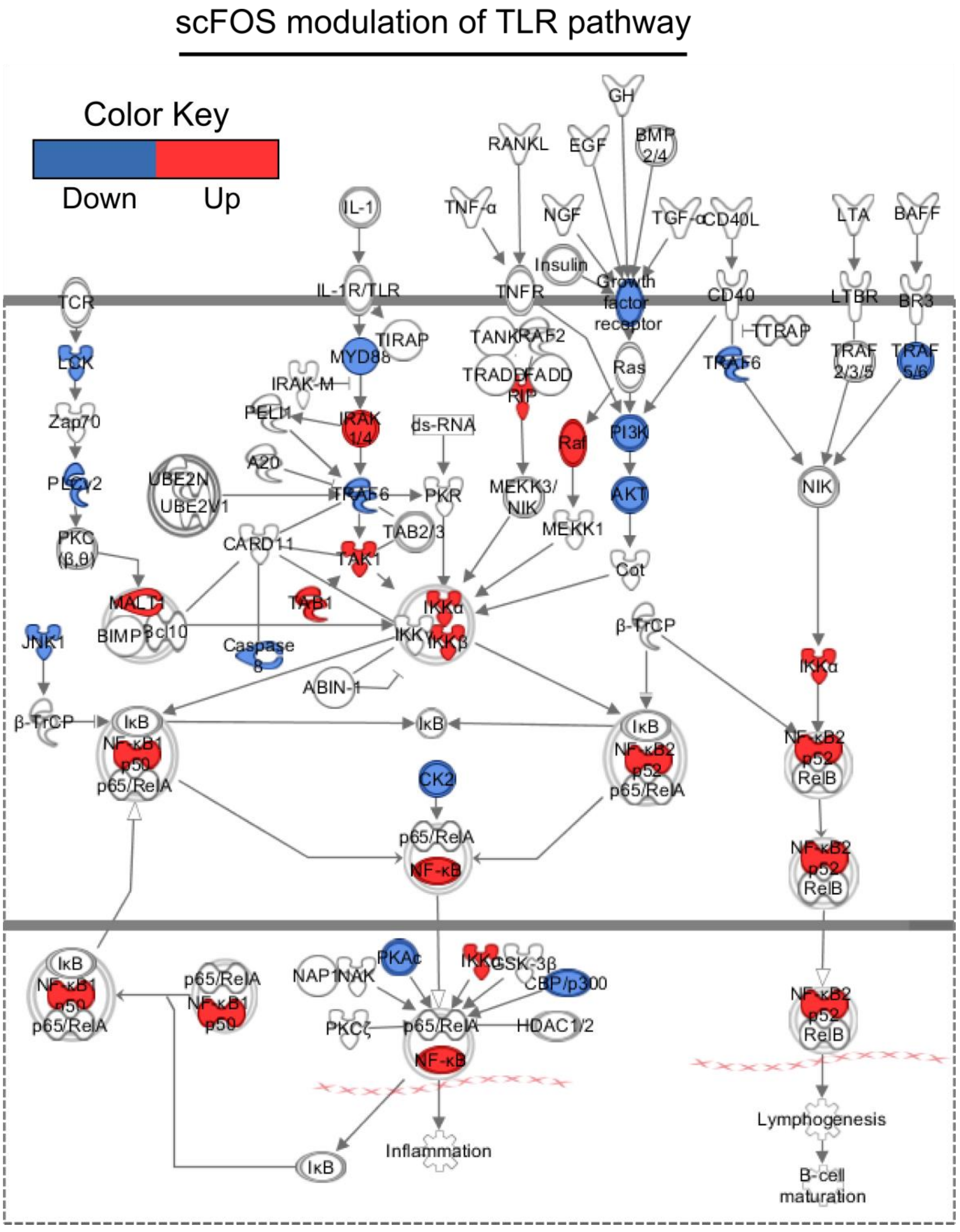

Supplementary Figure 2. Pathway visualization of kinases modulated by scFOS in the TLR signalling pathways was generated using Ingenuity Pathway Analysis (IPA).

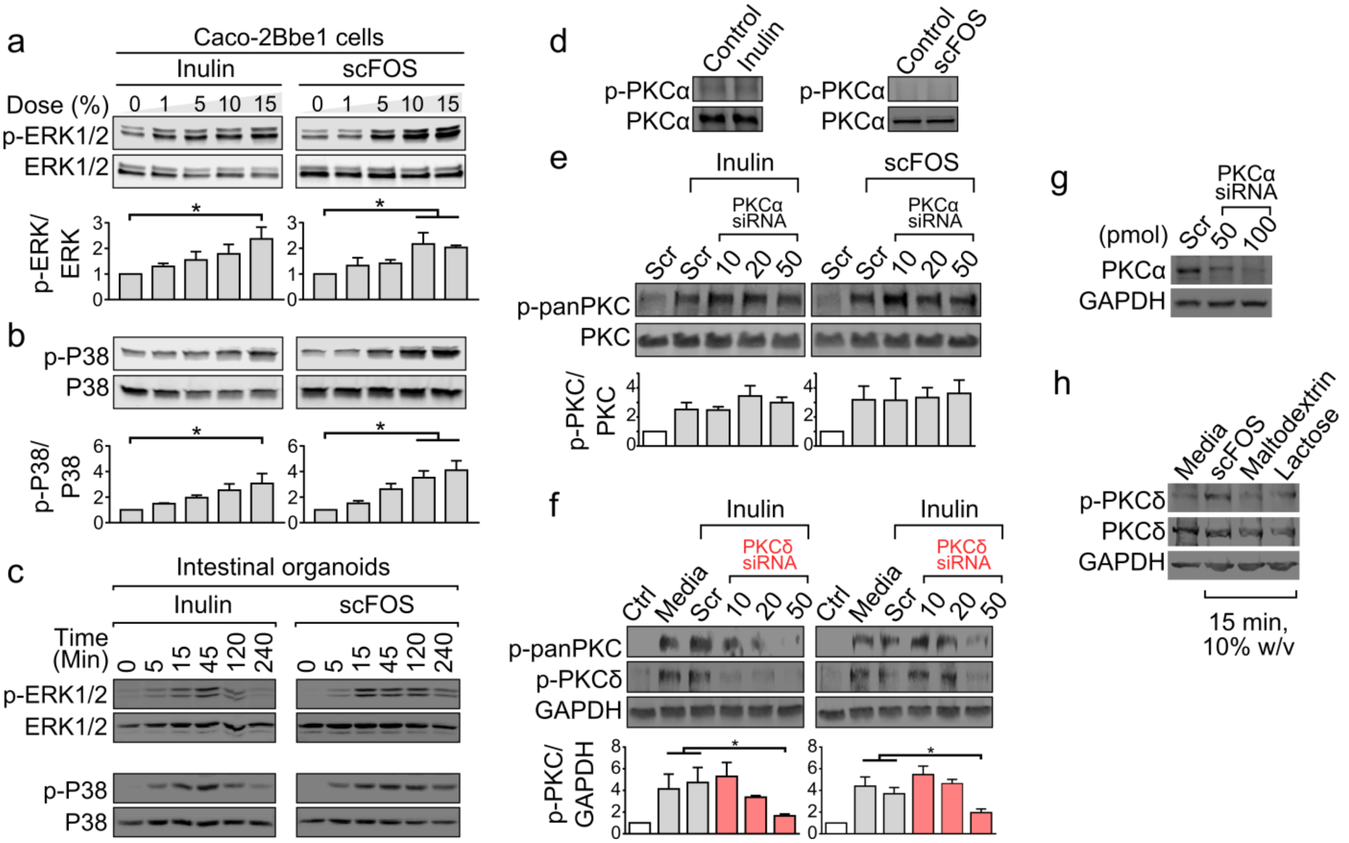

Supplementary Figure 3. Host signaling response to prebiotic inulin and scFOS. (**a-b**) Caco-2Bbe1 monolayers were treated with inulin or scFOS (0-15% w/v) for 15 min and immunoblotted for ERK1/2 and P38 MAPKs (n=4). (**c**) Intestinal organoids were grown as 2D monolayers were incubated with inulin or scFOS (10% w/v) for the specified duration and blotted for ERK1/2 and P38 MAPK phosphorylation (n=3). (**d**) Exposure to either inulin or scFOS for 15 min did not induce PKCα phosphorylation (n=3). (**e**) Caco-2Bbe1 monolayers transfected with PKCα siRNA at 10, 20 and 50 pmol (48 h) and then stimulated with either inulin or scFOS for 15 min (n=4). (**f**) Caco-2Bbe1 cells treated with PKCδ siRNA at 10, 20 and 50 pmol (48 h) were exposed to either inulin or scFOS for 15 min (n=4). (**g**) Caco-2Bbe1 cells were transfected with PKCα siRNA and knockdown was validated using western blotting. (**h**) Caco-2Bbe1 cells were treated with media alone, media with 10% scFOS, 10% maltodextrin or 10% lactose for 15 minutes and blotted for PKCδ activation (n=2). All values are represented as means, ± SEM. ANOVA with Bonferonni post-hoc testing, * P<0.05.

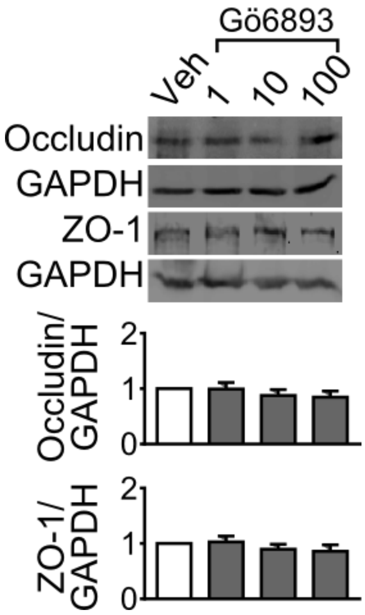

Supplementary Figure 4. Host TJ levels in response to Gö6893. Caco-2Bbe1 cells were treated with Gö6893 for 24 h in the concentrations 1, 10 and 100 nM and then immunoblotting undertaken to determine levels of ZO-1 and occludin (n=4).

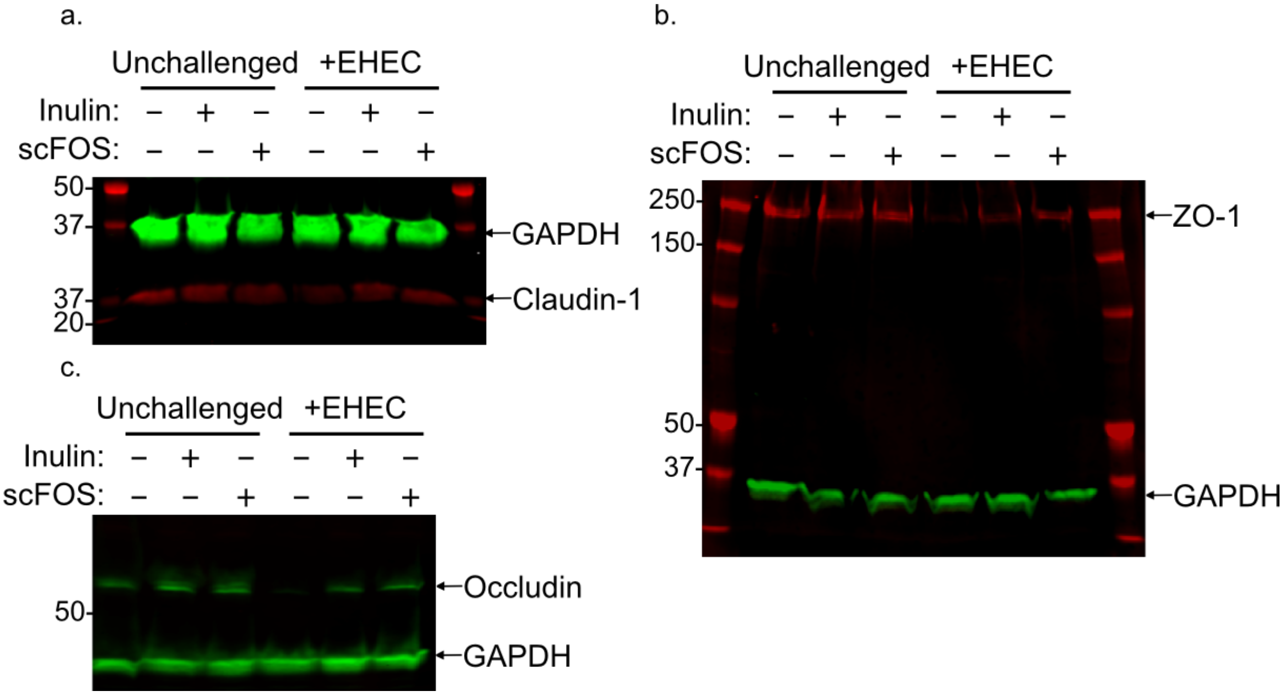

Supplementary Figure 5. Original immunoblots used to crop the gel bands for Figure 2a-c.

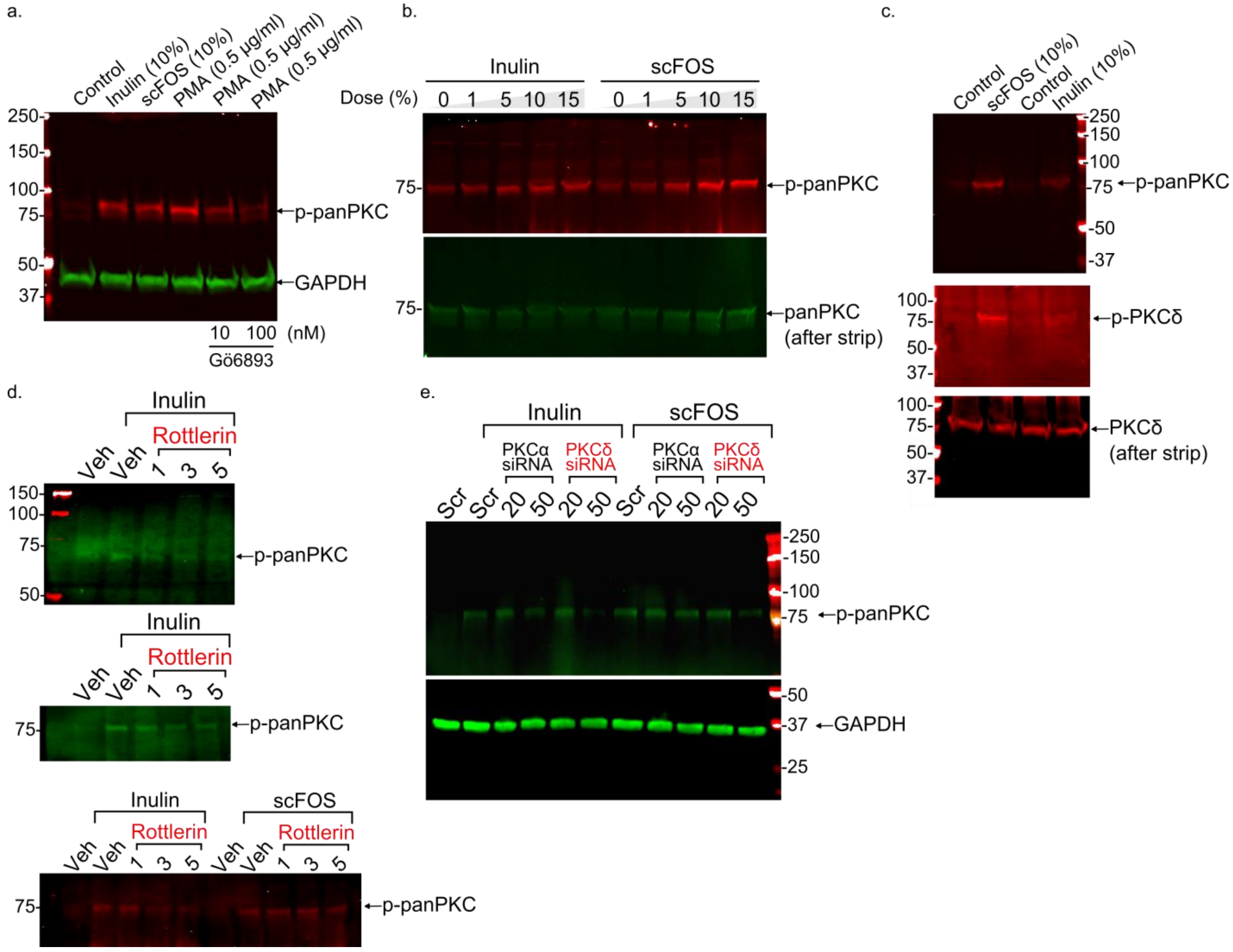

Supplementary Figure 6. (a) Phorbol 12-myristate 13-acetate (PMA), an inducer of PKC phosphorylation, was used as a positive control to test the specificity of anti-phospho-panPKC antibody in detecting PKC phosphorylation events. (**b-e**) Original immunoblots used to crop the gel bands for Figure 4b-e.

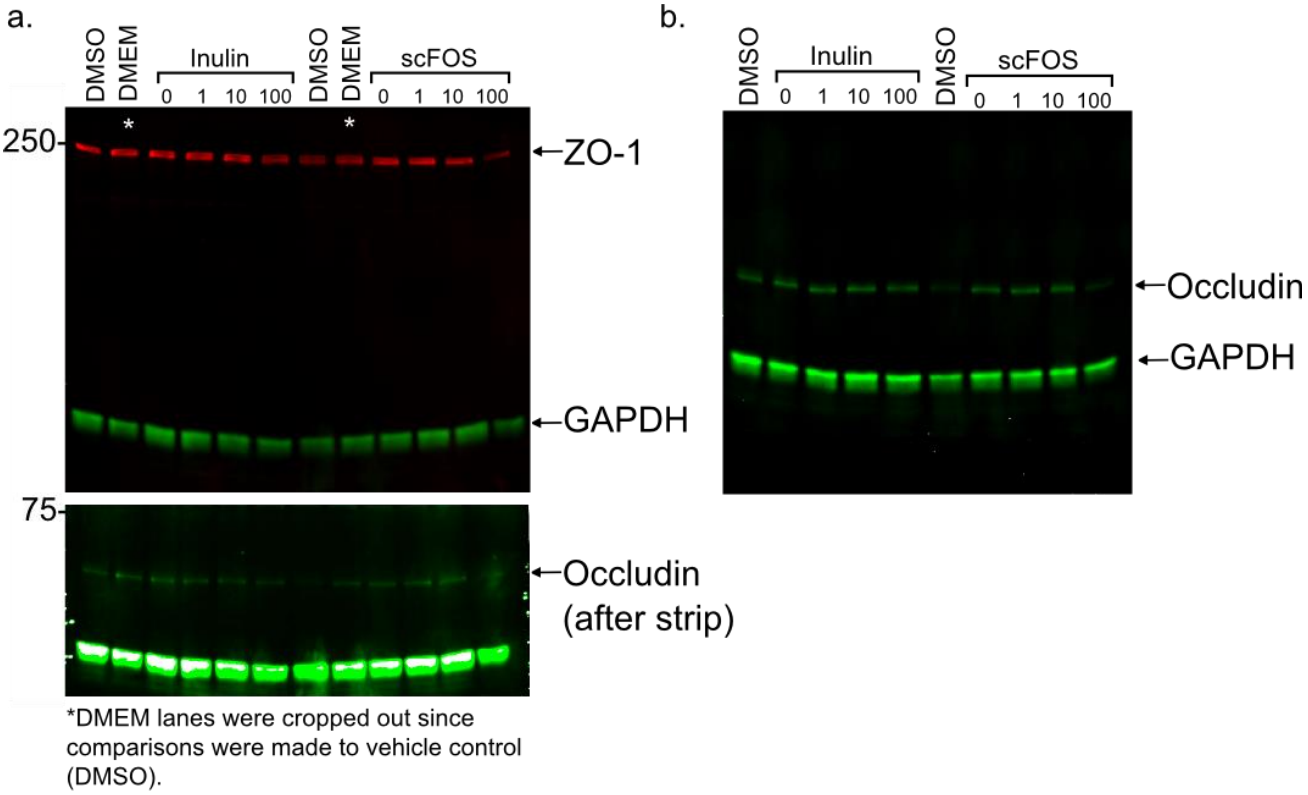

Supplementary Figure 7. Original immunoblots used to crop the gel bands for Figure 5e. * indicates the lanes removed to splice together the adjacent regions.
